# Frequency-resolved analysis of coherent oscillations of local cerebral blood volume, measured with near-infrared spectroscopy, and systemic arterial pressure in healthy human subjects

**DOI:** 10.1371/journal.pone.0211710

**Published:** 2019-02-12

**Authors:** Kristen Tgavalekos, Thao Pham, Nishanth Krishnamurthy, Angelo Sassaroli, Sergio Fantini

**Affiliations:** Department of Biomedical Engineering, Tufts University, Medford, Massachusetts, United States of America; Boston University, UNITED STATES

## Abstract

We report a study on twenty-two healthy human subjects of the dynamic relationship between cerebral hemoglobin concentration ([HbT]), measured with near-infrared spectroscopy (NIRS) in the prefrontal cortex, and systemic arterial blood pressure (ABP), measured with finger plethysmography. [HbT] is a measure of local cerebral blood volume (CBV). We induced hemodynamic oscillations at discrete frequencies in the range 0.04–0.20 Hz with cyclic inflation and deflation of pneumatic cuffs wrapped around the subject’s thighs. We modeled the transfer function of ABP and [HbT] in terms of effective arterial (*K*^(*a*)^) and venous (*K*^(*v*)^) compliances, and a cerebral autoregulation time constant (*τ*^(AR)^). The mean values (± standard errors) of these parameters across the twenty-two subjects were *K*^(*a*)^ = 0.01 ± 0.01 μM/mmHg, *K*^(*v*)^ = 0.09 ± 0.05 μM/mmHg, and *τ*^(AR)^ = 2.2 ± 1.3 s. Spatially resolved measurements in a subset of eight subjects reveal a spatial variability of these parameters that may exceed the inter-subject variability at a set location. This study sheds some light onto the role that ABP and cerebral blood flow (CBF) play in the dynamics of [HbT] measured with NIRS, and paves the way for new non-invasive optical studies of cerebral blood flow and cerebral autoregulation.

## 1. Introduction

Oscillations of systemic arterial blood pressure (ABP) drive hemodynamic changes in the body’s macro- and microvasculature, elicit local responses in the peripheral circulation, and induce oscillations in the cerebral blood volume (CBV), which is the focus of this work. The dynamics between systemic ABP and local CBV reflect a combination of effects due to passive vascular compliance and active vascular reactivity, which in turn elicit dynamic changes in cerebral blood flow (CBF). The passive response is determined by the mechanical properties of blood vessel walls. The active vascular reactivity is responsible for cerebral autoregulation (AR), which maintains a relatively stable CBF despite changes in cerebral perfusion pressure (CPP), defined as the difference between ABP and intracranial pressure (ICP). AR plays an important role in the CBF response to ABP oscillations. There has been an increasing interest in using metrics of AR to assess cerebrovascular health or to guide therapy at the bedside [1]. The characterization of the dynamic relationship between CBV and ABP may help discriminate the passive and active mechanisms of the CBV response to ABP changes to better assess and characterize AR.

Near-infrared spectroscopy (NIRS) is a technique that affords local measurements of CBV and features desirable properties such as non-invasiveness, safety, portability, and cost effectiveness. It is important to observe that the NIRS measurements of CBV are derived from measurements of the total concentration of hemoglobin in tissue ([HbT]), through a factor given by the concentration of hemoglobin in blood. In this way, [HbT] is a marker for CBV. However, not all blood vessels contribute equally to the NIRS signal, depending on their size and location. In particular, larger blood vessels may act as total absorbers of near-infrared light, so that NIRS measurements tend to reflect blood volume that is mostly associated with the microvasculature. Another important feature of NIRS is its capability to measure the concentration of both oxygenated and deoxygenated species of hemoglobin ([HbO_2_] and [Hb], respectively), which may be combined to derive their difference ([HbD] = [HbO_2_] − [Hb]) and the oxygen saturation of hemoglobin (StO_2_ = [HbO_2_]/[HbT]), the latter being sometimes referred to as tissue oxygenation index (TOI). Over the years, a number of NIRS studies have investigated some aspects of the dynamic relationship between ABP and NIRS-measured quantities, as we describe below.

In some approaches, NIRS measures have been considered as surrogates of specific physiological quantities such as blood flow, ICP, or cerebrovascular pressure reactivity (CPR), defined as the ability of vascular smooth muscle to respond to changes in transmural pressure. For example, the hemoglobin volume index (HVx), defined as the correlation coefficient between fluctuations in [HbT] and ABP, has been proposed as a non-invasive alternative to a measure of CPR, the pressure reactivity index (PRx), which is given by the correlation coefficient between ICP and ABP [2–4]. Both HVx and PRx are defined by considering pressure and hemodynamic fluctuations in the low frequency range (<0.05 Hz). PRx has shown value as a diagnostic index; for example, a greater or more positive PRx (indicative of a more pressure passive vasculature) has been associated with worse outcomes in head-injured patients [5]. PRx has also shown promise in predicting the lower limit of AR [2]. The relationship between CPR and AR is quite complex [2,6]. For example, Lee *et al*. [2] noted that in most physiological cases, the limits of CPR are wider than the limits of AR, meaning that the range of blood pressure over which the vasculature is responsive is broader than the one over which blood flow is regulated and stays relatively constant. In patients with traumatic brain injury, the correlation between CPR and AR was found to be moderate, but statistically significant [7].

A number of NIRS studies have assessed AR by co-registering dynamic traces of ABP and either TOI [8–11] or [HbD] [12,13], following similar approaches as the ones used to compute PRx or HVx. Typically, slow hemodynamics (over time scales > 10–20 s or frequencies < 0.05–0.10 Hz) were considered, and high levels of co-variation of ABP and TOI or HbD were associated with poor AR.

NIRS studies of AR have found inspiration from methods used in transcranial Doppler ultrasound (TCD), such as transfer function analysis (TFA) to determine phase and gain [14–17], or coherence analysis [18–21] of ABP (considered as input) and one of the NIRS measures (considered as output). The reason to consider the phase between ABP and hemodynamic oscillations is that TCD studies have found it to be a robust indicator of intact vs. impaired AR. Low frequency oscillations (≤0.1 Hz) of blood flow velocity should lead ABP oscillations for an intact AR, while they are approximately in phase for impaired AR. Even though NIRS cannot measure blood flow velocity directly, its measurements (especially tissue saturation and [HbD] [9,10]) are closely related to it. A few NIRS studies have investigated the phase relationship between ABP and NIRS parameters. Obrig *et al*. analyzed spontaneous oscillations and they found that the relative phase of [HbO_2_] and ABP decreased from slightly positive values (~10°-20°; i.e. [HbO_2_] leading ABP) at ~0.02 Hz to slightly negative values (~ -20°; i.e. [HbO_2_] lagging ABP) at 0.10 Hz [14]. TFA between ABP and NIRS measures was also applied to induced oscillations of ABP during paced breathing protocol at 0.1 Hz, and the relative phase of ABP and NIRS parameters was found to correlate with the loss of AR in patients affected by carotid artery stenosis [15,16].

Non stationary metrics of correlation between ABP and NIRS parameters, based on wavelet transform (WT), have also been used for studying AR [22–25]. The use of non-stationary methods of data analysis, like WT or short time Fourier transform (STFT), are more suitable for studying spontaneous and induced oscillations when the investigated hemodynamics feature a frequency spectrum that is time dependent. Wavelet parameters and derived metrics that have been considered include wavelet coherence (WCOH), wavelet phase (WPH), wavelet phase coherence (WPHC), and wavelet cross correlation (WCC). While WCOH and WCC are metrics that are affected by both amplitude and phase relationships, WPCOH is a metric of synchronization based solely on the relative phase. In the work of Papademetriou *et al*. WCC and WPH between ABP and [HbO_2_] were studied in a group of newborn infants on life support (extracorporeal membrane oxygenation, ECMO), during seven levels of flow from the support system [23]. These metrics were studied in three frequency ranges including a Mayer waves range 0.06–0.13 Hz. At a group level (*n* = 6) the authors found that, with the exception of one NIRS channel and one ECMO flow, the oscillations of ABP always lead the oscillations of [HbO_2_] in the Mayer waves range. Rowley *et al*. studied WCC, WPH and WPCOH between ABP and [HbO_2_] in patients suffering from autonomic failure during posture changes (head up tilt) [24]. Significant changes in the wavelet scale where the maximum WCC occurred was observed in patients, but not in a group of healthy controls. Also, at a group level and at a frequency ~0.05 Hz, the patients showed a significant phase change between ABP and [HbO_2_] during posture change: ~11° at baseline, ~-34° during head up tilt, ~6° post tilt. On the contrary, the heathy controls, at frequencies in the range 0.07–0.13 Hz, showed an almost constant negative phase in the range -17°–-6° for the three blocks of the protocol. We recall that a positive phase between ABP and [HbO_2_] means that the oscillations of ABP are leading those of [HbO_2_]. It is also worth mentioning that except for patients during head up tilt, in all other cases the phase error bars were larger than the mean values. Tian *et al*. studied WCOH, WPH and WPCOH between ABP and tissue saturation on a cohort of infants suffering from hypoxic-ischemic encephalopathy [25], and found that significant coherence for in-phase (0°±45°) signals occurred at time scales <80 min. On the contrary, significant coherence for anti-phase (180°±45°) signals was found at time scale around 2.5 h. Both in-phase and anti-phase significant coherence were related to worse clinical outcome [25].

The works referenced above provide clear indications that the phase and amplitude relationships between ABP and cerebral NIRS signals contain diagnostic information for the non-invasive assessment and monitoring of AR.

In this study, we performed measurements and developed an analytical model to investigate the frequency-resolved, dynamic relationship between local cerebral blood volume (i.e. [HbT] measured with NIRS) and systemic ABP. We measured 22 healthy subjects to characterize the relationship between [HbT] and ABP over multiple frequencies in the range 0.04–0.20 Hz. We used pneumatic thigh cuffs to induce controlled MAP oscillations to drive cerebral blood volume oscillations at a given frequency. We also implemented a time-varying approach for coherence analysis with a sliding short time Fourier transform (STFT). This enabled us to quantify coherence between [HbT] and ABP for thigh-cuff-induced oscillations as well as spontaneous oscillations, thus resulting in measurements of the transfer function at several frequencies beyond the ones specifically induced by the thigh cuffs.

We propose an analytical model for the phase and amplitude relationships between [HbT] and ABP. This model results in a transfer function for [HbT] and ABP that consists of a constant term plus a frequency dependent term in the form of a first-order low pass filter. The physical interpretation of the constant term is an arterial compliance effect, which becomes dominant at high frequencies (>1 Hz), whereas the frequency-dependent term (which is dominant at low frequencies, <0.1 Hz) is assigned to cerebral blood flow effects on the venous compartment. Importantly, the time constant of the low pass filter characterizes the AR response and can be used as an AR metric. This model provides a framework for measurements of AR that are based on [HbT] dynamics driven by ABP. Because dynamic NIRS measurements of [HbT] typically feature a better signal-to-noise ratio than measurements of [Hb] and [HbO_2_], the approach described in this work may be more robust than methods that we have previously introduced to assess AR from dynamic measurements of [Hb] and [HbO_2_] [26–28].

## 2. Methods

### 2.1 Human subjects

Twenty-two healthy subjects (15 females; 7 males; age range: 21–51 years old) participated in the study. The Tufts University Institutional Review Board approved the experimental protocol, and the subjects provided written informed consent prior to the experiment. The data for nine of the female subjects (nn. 13–21) have been previously analyzed for a comparison of the relative phase and amplitude of oxyhemoglobin and deoxyhemoglobin concentration oscillations in brain and breast tissue [29].

### 2.2 Experimental protocol and data acquisition

**Fig 1:**
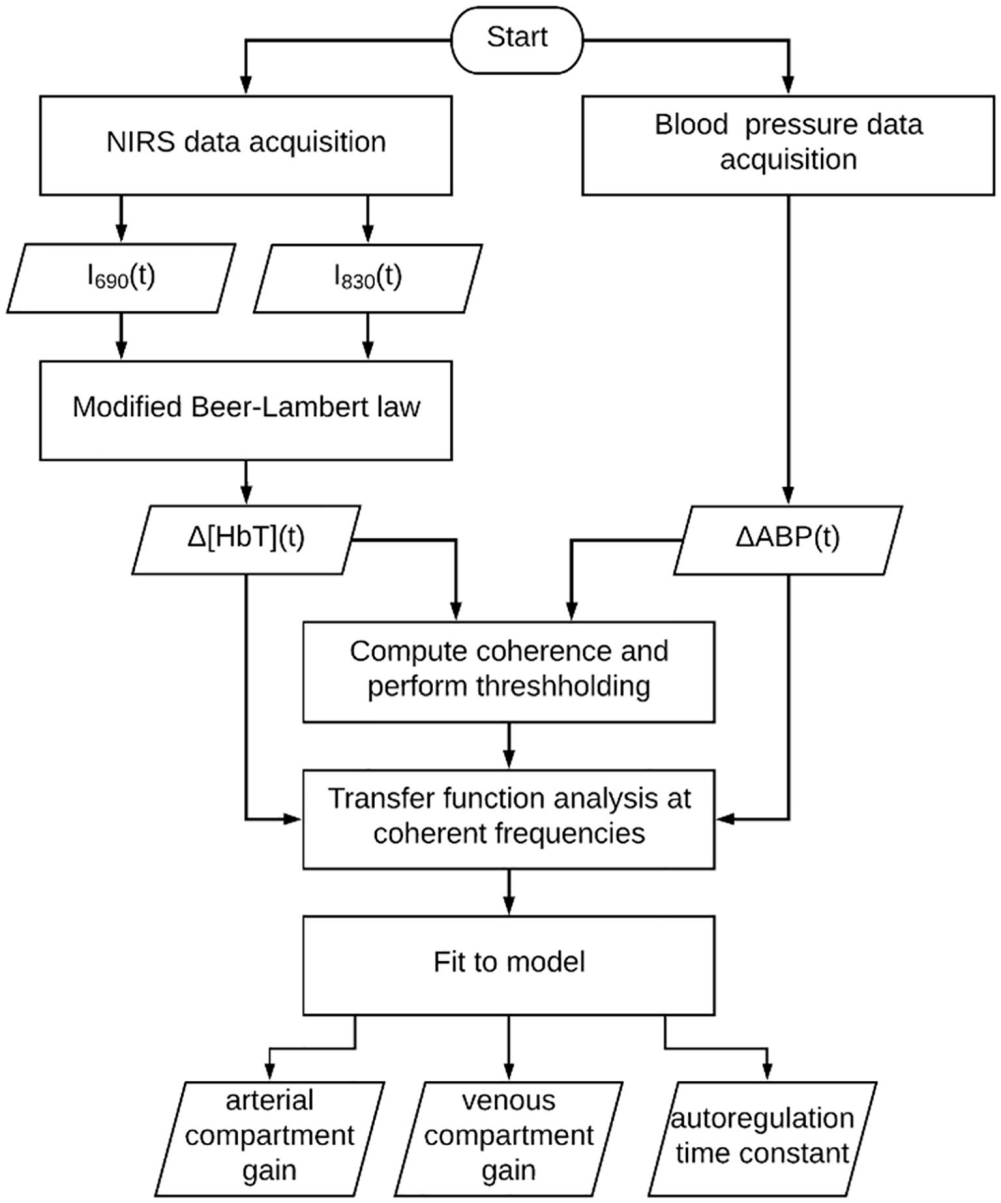
Flow chart of data collection and analysis. *I*_690_(*t*) and *I*_830_(*t*) are the light intensities detected at 690 nm and 830 nm, respectively. Δ[HbT](*t*) is the relative change in total hemoglobin concentration. ΔABP(*t*) is the change in arterial blood pressure relative to baseline.

Fig 1 is a flow chart to illustrate the framework for data collection and analysis. Each of the blocks in Fig 1 is described in the following text.

**Fig 2:**
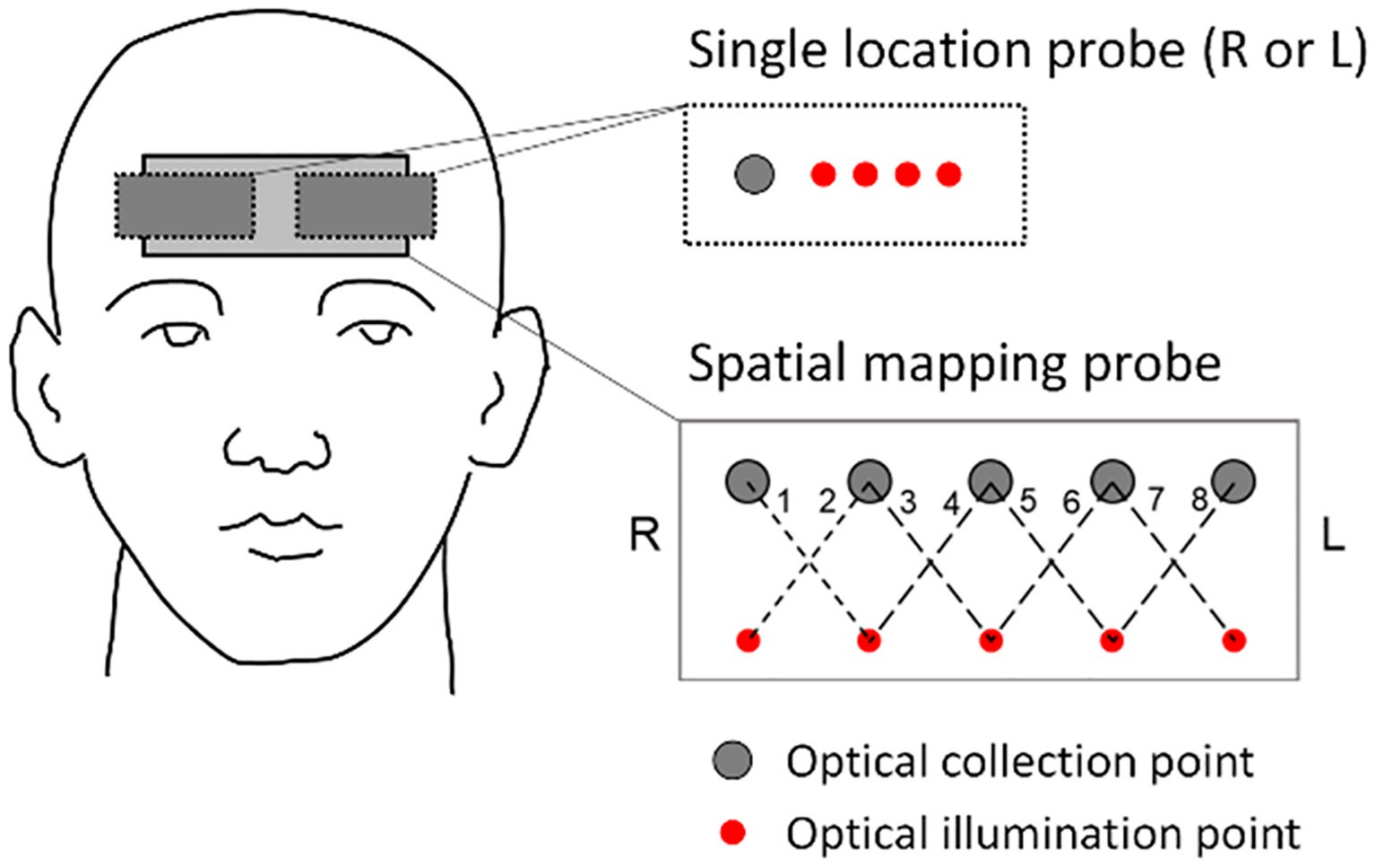
Configuration of the optical probes on the subject’s forehead. The single location probe is placed on either the right side (R: subjects 9–12) or left side (L: subjects 13–22) of the forehead, and features four source-detector separations of 2.0, 2.5, 3.0 and 3.5 cm. The spatial mapping probe (subjects 1–8) has eight single-distance channels (numbered 1–8 in the figure) at a source-detector distance of 3.5 cm, and realizes self-calibrated absolute measurements using source-detector distances of 2.5 and 3.5 cm. The optical illumination points represent the locations where light at 690 and 830 nm is delivered to tissue.

In each experiment, the subject sat on a chair with their feet on the floor or sat on a bed with their back at a ~30 degree angle and their legs parallel to the floor. A near-infrared spectroscopy (NIRS) probe was placed against the forehead [8 subjects (nn. 1–8): spatial mapping probe with eight source-detector channels; 14 subjects (nn. 9–22): single source-detector channel]. For details on the particular set up for each subject, see Table 1. Diagrams of the channel locations for the single channel probe and for the spatial mapping probe are shown in Fig 2. The single channel probe was a flexible commercial probe (ISS Inc., Champaign, Illinois). The spatial mapping probe was custom made from a 3D printed, flexible plastic frame with holes for source and detector optical fibers. The probe frame was surrounded with black silicone for subject comfort and to block room light. The optical probe was connected to a frequency-domain commercial NIRS instrument [10 subjects (nn. 1, 3–8, 10–12): OxiplexTS, ISS Inc., Champaign, Illinois; 12 subjects (nn. 2,9, 13–22): Imagent, ISS Inc. Champaign, Illinois]. The two instruments (OxiplexTS and Imagent) operate similarly, both employing fiber-optic coupled light sources (laser diodes) and optical detectors (photomultiplier tubes). Light at wavelengths of 690 and 830 nm was delivered to the subject’s forehead to probe the prefrontal brain cortex. The light intensity for each dual-wavelength pair of sources (*I*_690_(*t*) and *I*_830_(*t*)) was collected at a distance of 3.5 cm from the illumination point on the subject’s forehead. The modified-Beer Lambert law was used to compute relative changes in oxyhemoglobin (Δ[HbO_2_]), deoxyhemoglobin (Δ[Hb]), and total hemoglobin concentrations (Δ[HbT]) in units of microMolar (μM). The relative differential path length factor and baseline optical properties at 690 and 830 nm were determined from frequency-domain data either by a multi-distance approach (for single probe: source-detector distances of 2.0, 2.5, 3.0, and 3.5 cm) [30] or a self-calibrating approach (for the spatial-mapping probe: source-detector distances of 2.5 and 3.5 cm) [31]. Extinction coefficients for oxyhemoglobin and deoxyhemoglobin were taken from the literature [32].

**Table 1:** Summary of subjects.

Subject #	Sex	Age (y)	Spaced frequencies (Hz)	Consecutive frequencies (chirp-like) (Hz)	Number of channels	Subject position
1	F	27	-	0.040, 0.067, 0.093, 0.120, 0.146, 0.173, 0.200	8	Chair
2	M	27	0.040, 0.067, 0.093, 0.120, 0.146			
3	M	27				
4	F	28				Bed
5	M	31	0.040, 0.067, 0.093, 0.120, 0.146, 0.173, 0.200			
6	M	32				
7	F	26				
8	F	27	0.042, 0.056, 0.069, 0.083, 0.097, 0.111, 0.125	-		Chair
9	F	33	0.050, 0.063, 0.076, 0.090	0.050, 0.063, 0.076, 0.090	1 (R)	
10	M	28	0.040, 0.067, 0.093, 0.120, 0.146, 0.173, 0.200	0.040, 0.067, 0.093, 0.120, 0.146, 0.173, 0.200		
11	M	21				
12	F	24	0.040, 0.093, 0.146, 0.200			
13	F	30	0.046, 0.056, 0.063, 0.071, 0.083	-	1 (L)	
14	F	25				
15	F	32				
16	F	24				
17	F	25				
18	F	25				
19	F	27				
20	F	24				
21	F	27				
22	M	51	0.036, 0.058, 0.080, 0.102, 0.124, 0.146, 0.168, 0.190			

Column 1: subject number; column 2: sex (F or M); column 3: age; column 4: frequencies of oscillations that had time spacing in between; column 5: frequencies of oscillations that had no time spacing in between such that they were chirp-like; column 6: number of channels/side (R-right; L-left); column 7: subject position (on a chair with feet on the floor, or on a bed with legs parallel to the floor). Data for subjects 13–21 were also reported in a different study to compare brain and breast hemodynamics [29].

To induce MAP oscillations, pneumatic cuffs were placed around both subject’s thighs and were connected to a cuff inflation system (E-20 Rapid Cuff Inflation System, D.E. Hokanson, Inc., Bellevue, Washington). A microcontroller was connected to the regulator of the inflation system via a 1/4 inch stereo phone plug in order to set customized rates of inflation and deflation (Arduino Uno R3). Maximum inflation pressure of the cuffs was at least 180 mmHg, therefore greater than systolic blood pressure, in order to induce arterial occlusion. The air pressure in the thigh cuffs was continuously monitored with a digital manometer (Series 626 Pressure Transmitter, Dwyer Instruments, Inc., Michigan City, Indiana). Continuous arterial blood pressure (ABP) was recorded with a beat-to-beat blood pressure monitoring system (NIBP100D, BIOPAC Systems, Inc., Goleta, California). A pulse oximeter measured the heart rate (OXY100E, BIOPAC systems, Inc, Goleta, California or Nellcor PM-1000, Nellcor Inc., Hayward, CA). All signals were recorded synchronously with a sample rate of at least 6.25 Hz.

The experiment duration was 20–60 minutes. In each experiment, the thigh cuffs were cyclically inflated and deflated at frequencies ranging from 0.04 Hz to 0.20 Hz in order to induce MAP oscillations that would drive coherent oscillations in cerebral hemodynamics. In one protocol, we induced oscillations at a set of frequencies with sequential 90 s epochs of oscillations at a given frequency followed by 90 s of baseline. Another protocol was a “chirp” like sequence where we also induced oscillations at a set of frequencies, but this time each frequency was applied for a duration of 6 periods of oscillation, without baseline periods in between each frequency. See Table 1 for the set of induced frequencies for each subject and each protocol. We think that the differences in our experimental protocols do not negatively impact our goal to investigate the frequency-resolved relationship between coherent ABP and [HbT] oscillations, which is assumed to be independent of the timing, duration, and physiological origin of ABP oscillations. Coherence between ABP and [HbT] was computed across the frequency range of interest, as described in the next section. Coherence thresholding enabled us to consider both induced and spontaneously occurring hemodynamic oscillations for transfer function analysis.

**Fig 3:**
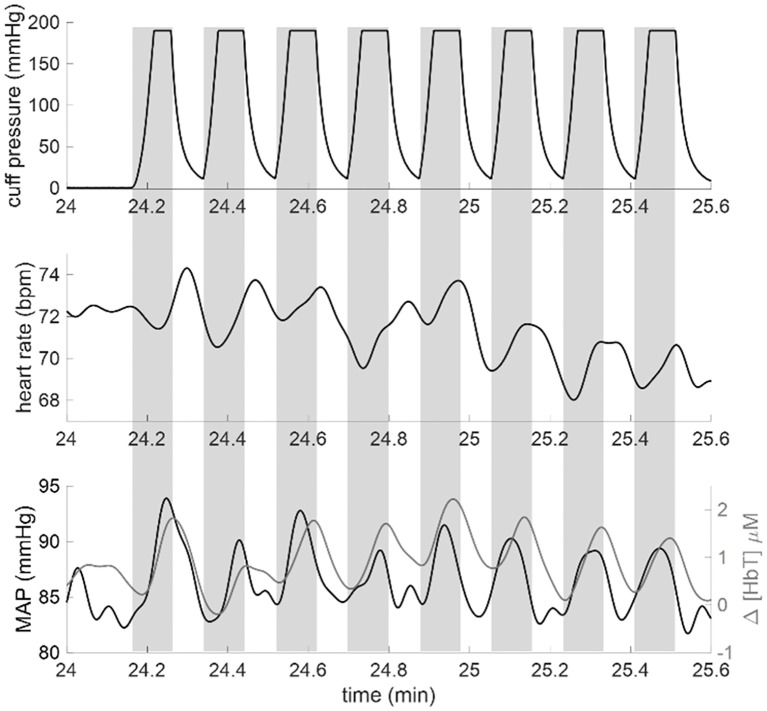
Example time traces of induced oscillations from Subject 5. Gray rectangles indicate the cuff inflation periods (from start of inflation to start of deflation). Top panel: Thigh cuff pressure measured with the manometer. Thigh cuffs were inflated for 11 seconds and deflated for 11 seconds to induce hemodynamic oscillations at a frequency of 0.093 Hz. Middle panel: Heart rate measured with the pulse oximeter in units of beats per minute (bpm). Bottom panel: mean arterial pressure (MAP) (black line) and total hemoglobin concentration changes (Δ[HbT], gray line). Signals in the middle panel and bottom panel have been low-pass filtered with a cut-off frequency of 0.25 Hz to suppress high-frequency noise and heart rate pulsations.

Fig 3 shows an example of the time traces collected for each experiment in order to convey how cuff pressure changes induced both systemic changes in MAP as well as local hemodynamic changes reflected in Δ[HbT]. The traces in Fig 3 are from the data set of subject 5 (channel 8) during the segment of the experiment where 0.093 Hz oscillations were induced. The top panel shows the thigh cuff pressure measured with the manometer. Gray rectangles indicate the cuff inflation periods (from start of inflation to start of deflation). The middle panel shows heart rate in beats per minute (bpm). The bottom panel shows the time traces for MAP (black line) and Δ[HbT] (gray line).

### 2.3 Time-varying coherence and coherence threshold

The mean value of ABP during baseline was subtracted from the ABP data in order to obtain relative changes of ABP in units of mmHg, notated as ΔABP. In order to perform transfer function analysis (TFA) between ABP and [HbT], we considered the coherence of ΔABP(*t*) and Δ[HbT](*t*) as a measure of their level of co-variation. Signals are coherent at a particular frequency when their relative amplitude and phase are stable at that frequency. The International Cerebral Autoregulation Research Network (CARNet) has developed guidelines for the standardization of coherence computation and transfer function analysis in this field [17]. In agreement with these guidelines, we performed no detrending, normalization, or filtering of the raw signals, we considered data segments longer than 100 s (specifically, 180 s), and we applied an anti-leakage window (specifically, a Hamming window) with 50% superposition of data segments. However, because in our study the various frequencies were induced sequentially, the measured signals were non-stationary and we needed to obtain time-resolved measurements of coherence spectra, as it will be explained in detail later in this section. In fact, ABP and [HbT] may have been coherent at a certain frequency during a particular temporal segment of the experiment but not necessarily for the entire experiment. In a recent study of stationary and non-stationary approaches for the analysis of covariation between ABP and [HbT], we argued that non stationary methods are more appropriate for spontaneous hemodynamics or for cases of induced hemodynamic oscillations during specific time intervals (as in the case of this work), when the phase locking of ABP and [HbT] might be temporary[33]. Therefore, in this work we have opted to compute the coherence between ABP and [HbT] with a time-varying approach based on the short time Fourier transform (STFT).

The mean squared coherence as a function of angular frequency ω and time *t*, MSC_ABP,[HbT]_(*ω*, *t*), is obtained as follows:

$${MSC}_{ABP,[HbT]}(\omega ,t)=\frac{{\left|P_{ABP,[HbT]}(\omega ,t)\right|}^{2}}{P_{ABP,ABP}(\omega ,t)P_{\left[HbT],[HbT\right]}(\omega ,t)},$$ (1)

where *P*_ABP,[HbT]_(*ω*, *t*) is the cross power spectral density between ABP and [HbT], *P*_ABP,ABP_(*ω*, *t*) is the power spectral density of ABP, and *P*_[HbT],[HbT]_(*ω*, *t*) is the power spectral density of [HbT]. Perfect linear coupling results in a coherence of 1, whereas absence of coupling results in a coherence of 0. We elected to perform the spectral analysis with the STFT based on Welch’s overlapped averaged periodogram method [34]. Our frequency range of interest in relation to cuff-induced hemodynamics was up to 0.20 Hz, but we extended it to the heart rate range (~1 Hz) to investigate pulsatile hemodynamics. The STFT was performed with a time resolution of 180 s and a frequency resolution of 0.0133 Hz. Each 180 s segment of data was divided into two windows with 50% overlap which were each multiplied by a Hamming window to reduce spectral leakage. The MATLAB function “mscohere” performed the coherence computation. The window for analysis was repeatedly shifted by 4 s in order to create a pseudo-continuous time trace of coherence for each frequency. The output of this procedure was an image of coherence, with time along the *x*-axis and frequency along the *y*-axis. Therefore, the differences in data processing between our study and the CARNet guidelines are as follows: in the CARNet guidelines the Hamming windows with 50% overlap are used to define spectral samples to be averaged for the entire data set (i.e. the spectral samples belong to the same population because the data are treated as stationary); on the contrary, in our study only two spectral samples (that are averaged every 180 s) are considered to belong to the same population (i.e. the data are treated as non stationary).

An alternative non-stationary approach is based on wavelet analysis. The performance of the STFT and wavelet approaches are similar, although the wavelet approach has a more straightforward implementation of a frequency-varying time resolution [35]. For our relatively narrow frequency range of interest, we opted for the STFT approach because frequency-varying time resolution was not needed.

To compute a threshold for significant coherence, we used the following approach [33]. Two sequences of random numbers were generated to simulate time traces of incoherent I_690_(*t*) and I_830_(*t*). The modified Beer Lambert law translated these intensity time traces into temporal traces of total hemoglobin concentration (Δ[HbT]). Another sequence of independent random numbers was generated to simulate ΔABP. Our approach for coherence analysis was applied to these independent sets of random numbers 1000 times to generate the null statistical distribution for coherence. We found that the 5% critical value of coherence between ABP and [HbT] was 0.79, so we selected this value as our threshold for significance. We applied this threshold to the image of coherence, and we defined a binary image, where the pixels above threshold were assigned the value of 1 and those below threshold were assigned the value of 0.

Following thresholding of the coherence image, we determined the connected components in the binary image (MATLAB function “bwconncomp”) which considers adjacent pixels as part of the same group. We computed the area of each separate group of connected pixels which we call clusters. We expected coherence between the two signals to be maintained for at least several seconds, so that single pixels or small clusters of pixels passing the threshold were assigned to noise. A second set of data for generating the null statistics for coherent cluster size was created with another run of 1000 random independent time traces of ΔABP and Δ[HbT]. Upon thresholding with the selected value of 0.79, the size of each coherence region within the image was recorded. We computed that regions greater than 13 (25) pixels had a 5% (0.2%) chance of being designated as coherent by chance. In the analysis of the experimental data, coherence was computed between ΔABP and Δ[HbT]. We created a binary image with 1’s indicating pixels that exceeded the coherence threshold. Then the size of coherent clusters was computed, and pixels in clusters whose size was less than 25 pixels were set to 0 in the binary image. We heuristically found that the threshold of 25 pixels, with a significance level of 0.2%, provided estimates of transfer functions with less variability than a threshold of 13 pixels without eliminating too many clusters. After the size thresholding, some single pixels protruded at the edges of clusters, and we assigned them to noise. A filter was applied to the binary coherence image to eliminate protruding pixels.

### 2.4 Transfer function analysis

The time-varying transfer function between ABP and [HbT] was computed with the same time resolution, frequency resolution, and windowing approach as the coherence computation. The MATLAB function “tfestimate” was used for this portion of the analysis. The transfer function over frequency and time is written as follows:

$$H_{ABP,[HbT]}(\omega ,t)=\frac{P_{ABP,[HbT]}(\omega ,t)}{P_{ABP,ABP}(\omega ,t)},$$ (2)

where the magnitude of the transfer function represents the relative change in μM of total hemoglobin concentration to mmHg of arterial blood pressure. The phase of the transfer function is the phase of [HbT] oscillations relative to the phase of ABP oscillations. [HbT] oscillations lead ABP oscillations when this phase difference is positive, while they lag ABP oscillations when this phase difference is negative.

The time-frequency transfer function, at pixels that passed the thresholding condition, was averaged over time with the assumption that each coherent pixel in time is a sample of the same transfer function. The time-averaged transfer function between ABP and [HbT] as a function of frequency is:

$$H_{ABP,[HbT]}(\omega )=\frac{\langle P_{ABP,[HbT]}(\omega ,t)\rangle }{\langle P_{ABP,ABP}(\omega ,t)\rangle }.$$ (3)

### 2.5 Dynamic model of blood volume response to arterial blood pressure changes

Blood volume changes result from two possible effects: vascular dilation/constriction (i.e. cross sectional changes of blood vessels) and vascular recruitment/collapse (i.e. opening of new vessels or closing of existing ones). Vascular recruitment/collapse may occur at the capillary level, and it is a common occurrence in skeletal muscle but not in brain tissue [36], so that we neglect it here. We also neglect cerebral capillary dilation or constriction even though it may happen to some extent [37,38]. We are then left to considering vascular dilation or constriction in the arterial and venous compartments.

Changes in arterial blood pressure, ΔABP(*t*), and therefore in arterial transmural pressure, directly result in changes in arterial blood volume, ΔCBV^(*a*)^(*t*), according to a factor, *C*^(*a*)^, that is related to an effective arterial compliance of all arterial vessels that are within the optically probed volume:

$${\Delta CBV}^{\left(a\right)}(t)=C^{\left(a\right)}\Delta ABP(t).$$ (4)

We consider the arterial compliance to be sufficiently small to allow us to neglect the difference between arterial inflow and outflow associated with the change in arterial blood volume (i.e. $F_{in}^{\left(a\right)}(t)\approx F_{out}^{\left(a\right)}(t)$, where *F*_in_ and *F*_out_ denote the inflow and outflow for the vascular compartment indicated by the superscript, (*a*) for the arterial compartment in this case). In the frequency domain, Eq (4) can be written as follows using phasor notation:

$${CBV}^{\left(a\right)}(\omega )=C^{\left(a\right)}ABP(\omega ),$$ (5)

where bold face **CBV**^(*a*)^ and **ABP** indicate phasors, which represent Fourier components at a given angular frequency *ω*. We use phasor notation to describe sinusoidal oscillations at a given angular frequency *ω*. The magnitude of the phasor is the amplitude of the oscillations at frequency *ω*, relative to the average value, and the argument of the phasor is the phase of the oscillations.

Even though arterial blood pressure dynamics typically induce associated venous blood pressure dynamics (for example, venous blood pressure does present some level of pulsation at the heartbeat frequency) the venous blood pressure is much lower than arterial blood pressure, so that we neglect direct effects of ABP on the venous transmural pressure, and thus onto venous blood volume. Instead, we consider venous blood volume dynamics resulting from cerebral blood flow changes, which are mostly driven by ABP changes in this study. Therefore, rather than being directly driven by ABP, we consider venous blood volume changes to be mediated by CBF changes. The arterial outflow acts as the capillary inflow (i.e. $F_{out}^{\left(a\right)}(t)=F_{in}^{\left(c\right)}(t)$) and, because we neglect changes in capillary blood volume, $F_{in}^{\left(c\right)}(t)=F_{out}^{\left(c\right)}(t)=F_{in}^{\left(v\right)}(t)$, where $F_{in}^{\left(v\right)}(t)$ is the venous inflow. It is important to observe that, under these approximations, the temporal dynamics of blood flow in the arterial and capillary compartments, as well as at the entrance of the venous compartment, are considered to be approximately the same. Such common flow value can be identified with the cerebral blood flow, CBF (i.e. $CBF(t)≅F_{in}^{\left(a\right)}(t)≅F_{in}^{\left(c\right)}(t)≅F_{in}^{\left(v\right)}(t)$).

The venous cerebral blood volume, CBV^(*v*)^(*t*), defined as the venous blood volume per unit volume of tissue within the optically probed region, can change only for an imbalance between venous inflow, $F_{in}^{\left(v\right)}(t)$, and outflow, $F_{out}^{\left(v\right)}(t)$ (inflow and outflow are defined here as the volume of blood flowing in or out, respectively, of the venous compartment in the optically probed region per unit time, per unit volume of tissue). The dynamics of venous blood volume obey the equation:

$$F_{in}^{\left(v\right)}(t)-F_{out}^{\left(v\right)}(t)=\frac{d{CBV}^{\left(v\right)}(t)}{dt}.$$ (6)

In a situation of equilibrium, at time *t* = 0, we have $F_{in}^{\left(v\right)}(0)=F_{out}^{\left(v\right)}(0)=F_{0}^{\left(v\right)}$ and ${CBV}^{\left(v\right)}(0)={CBV}_{0}^{\left(v\right)}$. Dynamic changes relative to the equilibrium condition are written as follows: ${\Delta F}_{in}^{\left(v\right)}(t)=F_{in}^{\left(v\right)}(t)-F_{0}^{\left(v\right)},{\Delta F}_{out}^{\left(v\right)}(t)=F_{out}^{\left(v\right)}(t)-F_{0}^{\left(v\right)}$ and ${\Delta CBV}^{\left(v\right)}(t)={CBV}^{\left(v\right)}(t)-{CBV}_{0}^{\left(v\right)}$. With this notation, Eq (6) can be written in terms of venous flow and blood volume changes:

$${\Delta F}_{in}^{\left(v\right)}(t)-{\Delta F}_{out}^{\left(v\right)}(t)=\frac{d{\Delta CBV}^{\left(v\right)}(t)}{dt}.$$ (7)

If we make the further approximation that the venous compliance is large enough to accommodate any inflow changes through venous volume changes (so that ${\Delta F}_{out}^{\left(v\right)}(t)≅0$), and we recall that $CBF(t)≅F_{in}^{\left(v\right)}(t)$, we can write:

$$\Delta CBF(t)≅\frac{d{\Delta CBV}^{\left(v\right)}(t)}{dt}.$$ (8)

Because in the frequency domain a time derivative becomes a product by *iω*, the phasor version of Eq (8) is:

$${CBV}^{\left(v\right)}(\omega )=\frac{1}{i\omega }CBF(\omega ),$$ (9)

which states that the venous volume oscillations lag the driving flow oscillations by *π*/2 (since the phase of 1/*i* is −*π*/2) and are attenuated by a factor 1/*ω* as frequency increases. We note that this result of a *π*/2 phase lag of CBV with respect to CBF refers to the case of ideal sinusoidal oscillations.

As a result of dynamic cerebral autoregulation (AR), the temporal response of CBF to ABP changes can be described in terms of a high pass filter, through its impulse response function $h_{ABP,CBF}^{\left(AR\right)}(t)$:

$$\Delta CBF(t)=kh_{ABP,CBF}^{\left(AR\right)}(t)⊗\Delta ABP(t),$$ (10)

where *k* is a dimensional constant. Taking Eq (10) into account, and considering that in the Fourier domain a convolution product becomes a regular product, Eq (8) can be written in the frequency domain using phasor notation:

$$kH_{ABP,CBF}^{\left(AR\right)}(\omega )ABP(\omega )=i\omega {CBV}^{\left(v\right)}(\omega ),$$ (11)

where $H_{ABP,CBF}^{\left(AR\right)}(\omega )$ is the Fourier transform of $h_{ABP,CBF}^{\left(AR\right)}(t)$, or the transfer function of the autoregulation high pass filter. For the simple case of a resistor-capacitor (RC) high pass filter with time constant *τ*^(*AR*)^ (related to autoregulation), the impulse response function is $h_{ABP,CBF}^{\left(AR\right)}(t)=\delta (t)-\frac{1}{t}e^{-\frac{t}{\tau^{\left(AR\right)}}}$, and its Fourier transform is the transfer function $H_{ABP,CBF}^{\left(AR\right)}(\omega )=\frac{i\omega \tau^{\left(AR\right)}}{1+i\omega \tau^{\left(AR\right)}}$. In this case, Eq (11) yields:

$${CBV}^{\left(v\right)}(\omega )=\frac{k\tau^{\left(AR\right)}}{1+i\omega \tau^{\left(AR\right)}}ABP(\omega ).$$ (12)

We can now consider the total blood volume phasor, which results from the sum of the arterial and venous blood volume phasors given in Eqs (5) and (12):

$$CBV(\omega )={CBV}^{\left(a\right)}(\omega )+{CBV}^{\left(v\right)}(\omega )=(C^{\left(a\right)}+\frac{k\tau^{\left(AR\right)}}{1+i\omega \tau^{\left(AR\right)}})ABP(\omega ).$$ (13)

Because the total hemoglobin concentration, [HbT], measured with NIRS is given by the product ctHb CBV, where ctHb is the concentration of hemoglobin in blood, Eq (8) can be written as follows for the total hemoglobin concentration phasor:

$$[HbT](\omega )=(K^{\left(a\right)}+\frac{K^{\left(v\right)}}{1+i\omega \tau^{\left(AR\right)}})ABP(\omega ),$$ (14)

where *K*^(*a*)^ = ctHb *C*^(*a*)^ and *K*^(*v*)^ = ctHb *kτ*^(AR)^. Eq (14) represents the model used by us to fit the experimental data of [HbT] and ABP oscillations as a function of frequency.

To perform a fit to the model Eq 14) in the frequency domain, we implemented an algorithm, using the MATLAB function “fmincon,” to minimize the sum of the squared differences between the model prediction and the measured data, normalized by the error at each point. The range for *K*^(*a*)^ was set between 0.0001 and 0.2 μM/mmHg, the range for *K*^(*v*)^ was set between 0 and 10 μM/mmHg, and the range for *τ*^*AR*^ was set between 0 and 60 s. To compute the error on each parameter, we performed bootstrapping [39]. First, we calculated the residuals as the difference between the spectra computed with the model (by the fitted parameters) and the spectra obtained from the measured data. The spectrum obtained by the model fitting procedure was assumed to be a good representation of the data. The residuals were randomly sampled with replacement and added to the spectra computed by the model. Fitting was repeated to obtain a new set of parameters. This process was repeated 100 times to obtain a distribution of parameter values. The standard deviation of the 100 results, normalized by the square root of the number of iterations, was used to represent the error on the fitted parameters.

## 3. Results

### 3.1 Single location probe

**Fig 4:**
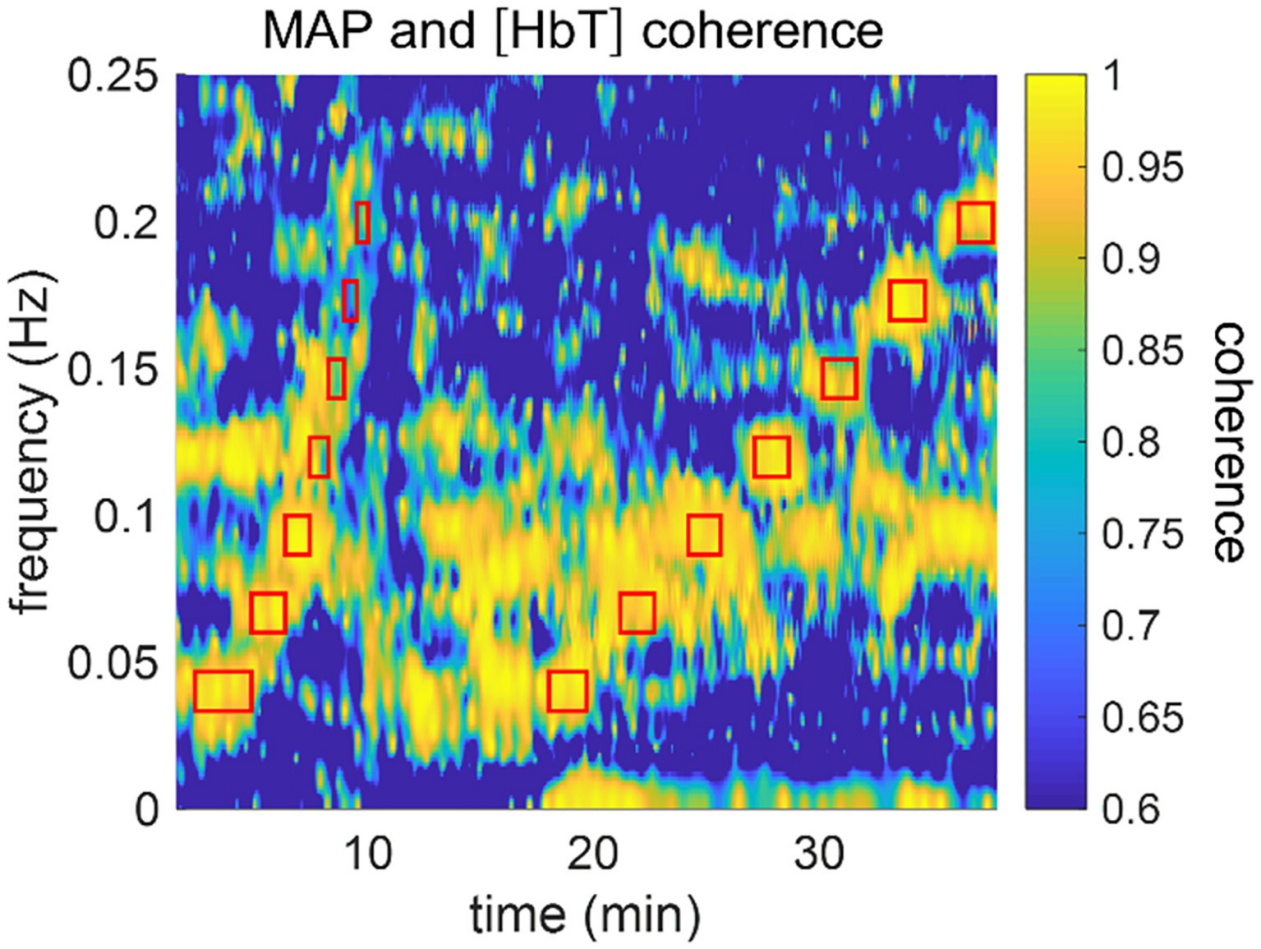
Time-frequency representation of coherence between [HbT] and MAP for subject 5, channel 8. Red rectangles indicate timing and frequency of oscillations induced with the pneumatic thigh cuffs. The group of rectangles on the left (times <10 min) are associated with the chirp-like protocol and the group of rectangles on the right (times >18 min) are associated with oscillations of equal duration and spacing over time.

The single location probe was placed on either the right side (subjects nn. 9–12) or left side (subjects nn. 13–22) of the subject’s forehead. For the subjects who wore the spatial mapping probe (subjects nn. 1–8), the left-most channel (channel 8 in Fig 2) was selected for single-location comparisons. Fig 4 shows the results of the time-frequency coherence computation for subject 5 during the entire protocol. Fig 4 was generated with a time window sliding by one sample at a time (i.e. 80–160 ms), but in our data analysis we used sliding windows by increments of 4 s (to decrease analysis time without affecting the results). The experiment duration was 40 min and the timing and frequencies of the induced thigh cuff oscillations are marked with red rectangles in Fig 4. The center of each rectangle indicates the center frequency and time. The top and bottom sides of the rectangles indicate the frequency band associated with the frequency resolution, whereas the left and right sides indicate the time interval in which any given frequency was induced. The first section of red rectangles (at times 0–10 min) indicates the chirp-like portion of the experiment where we induced six periods of oscillation at seven frequencies. The second section of red rectangles (at times 18–40 min) shows the set of sequential oscillations induced at seven frequencies for 90 s each, followed by 90 s of baseline. The coherence values in Fig 4 show that the cyclic inflations of thigh cuffs successfully enhanced coherence between ABP (or MAP, which is a low-pass filtered version of ABP) and [HbT]. In Fig 4, we use MAP rather than ABP for visualization purposes (MAP is less noisy). It is also visible in Fig 4 that [HbT] and MAP were spontaneously coherent for portions of the experiment in which the thigh cuffs were not cyclically inflated and deflated. This result is important because it shows that selected portions of spontaneous cerebral hemodynamic oscillations may be coherent with ABP, and the analysis represented by Fig 4 allows one to identify such portions in time and frequency.

**Fig 5:**
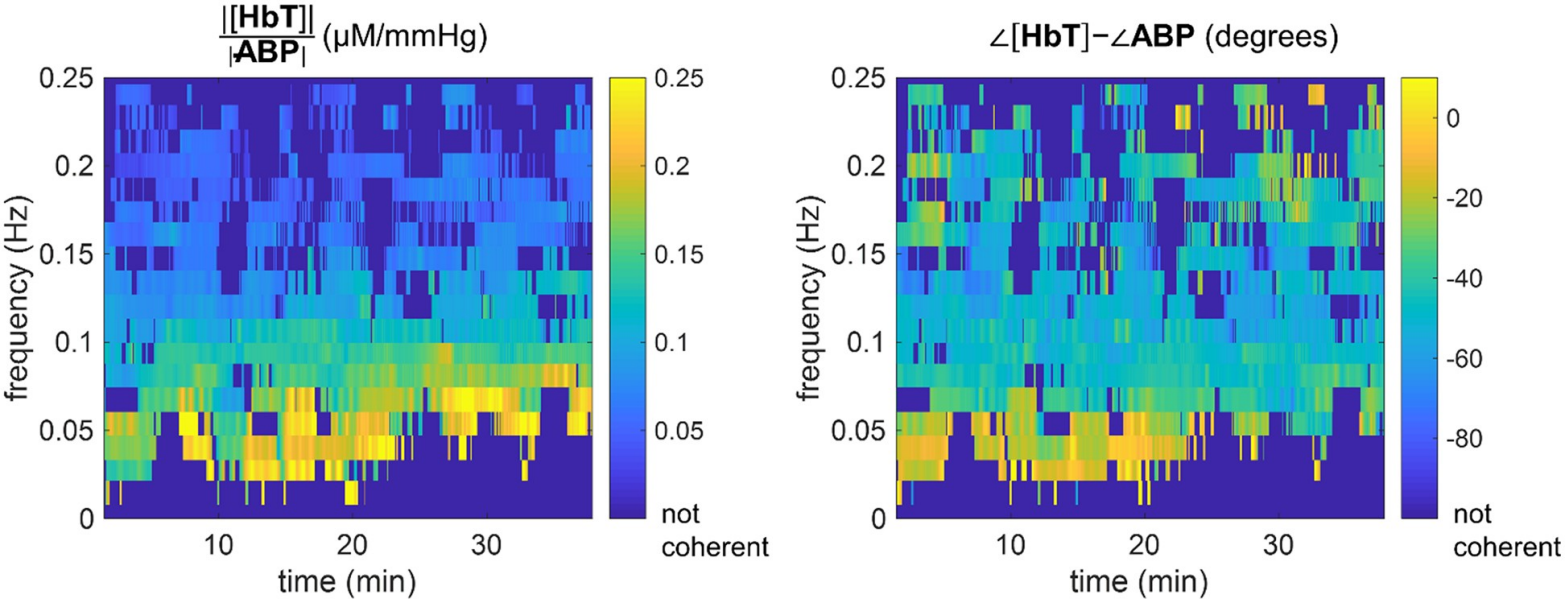
Transfer function analysis results for subject 5, channel 8. Left panel: Amplitude ratio |[HbT]|/|ABP|, i.e. |*H*_ABP,[HbT]_(*ω*, *t*)|, at the coherent pixels. Right panel: Phase difference ∠[HbT] − ∠ABP, i.e. Arg[*H*_ABP,[HbT]_(*ω*, *t*)], at the coherent pixels. Regions of the images which are dark blue did not pass the coherence threshold condition.

Fig 5 shows the results of the transfer function analysis for the data of subject 5, channel 8. The left and right panels of Fig 5 show the amplitude ratio and the phase difference, respectively, between [HbT] and ABP at the coherent frequencies. In Fig 5, the dark blue color indicates coherence values that do not exceed the threshold for significance (i.e., the 5% critical value of coherence). Fig 5 was generated with ABP, rather than MAP (as in Fig 4), to convey the actual time resolution of the analysis (rather than the interpolated version for visualization in Fig 4).

**Fig 6:**
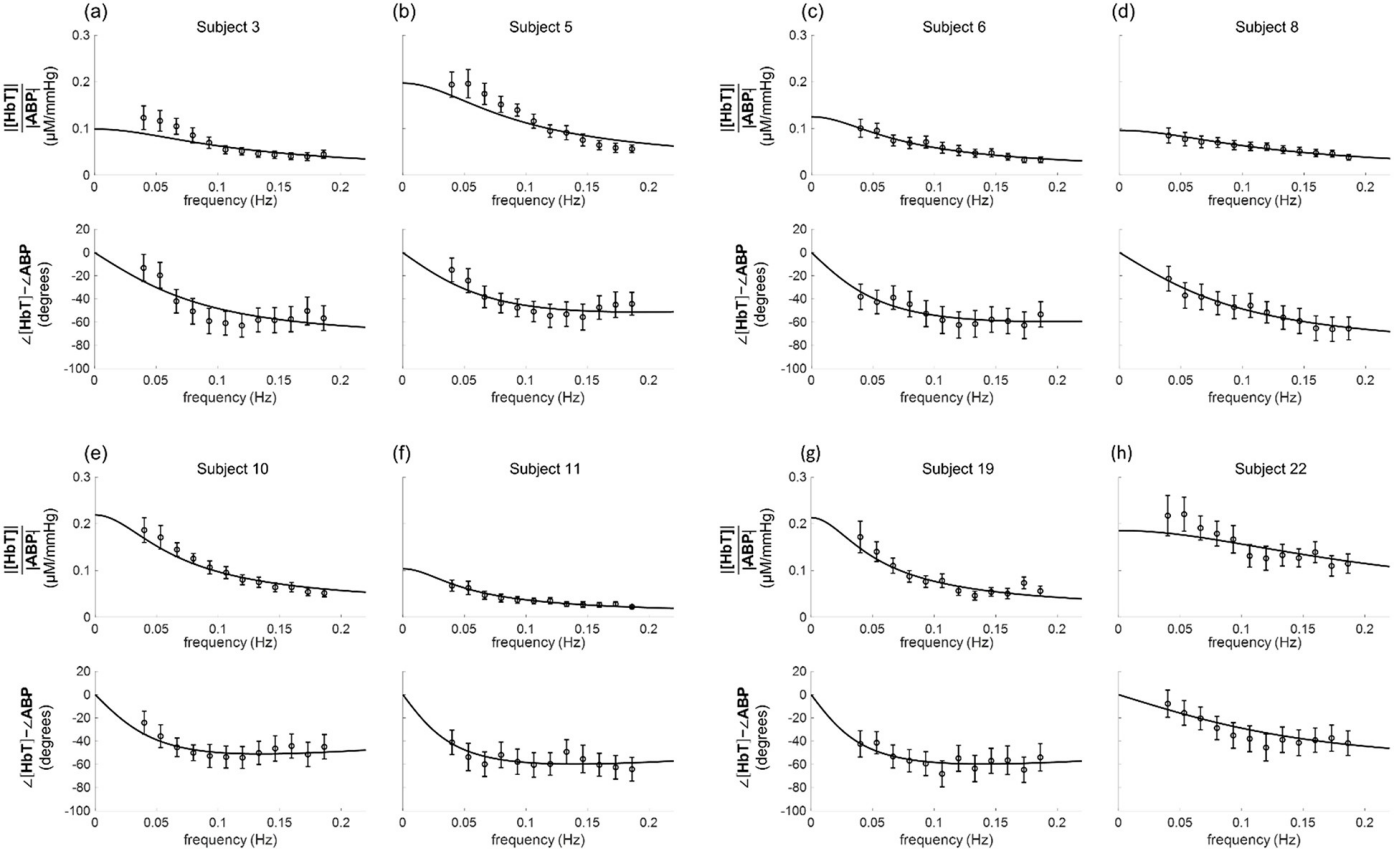
Experimental data and fitted transfer functions for the [HbT]-ABP amplitude ratio and phase difference of eight representative subjects. The symbols indicate the experimental data, the lines indicate the fitted transfer functions [represented by Eq (14)], and the error bars indicate the standard error of the mean.

Following the computation of *H*_ABP,[HbT]_(*ω*, *t*), the data were averaged over time to produce the spectra *H*_ABP,[HbT]_(*ω*), under the assumption of time invariance. The mean values and the standard error of the mean are indicated by the circles with error bars in Fig 6 for eight representative subjects: subjects nn. 3, 5 (also represented by the data in Figs 4 and 5), 6, 8, 10, 11, 19, and 22. Table 2 reports a summary of the **ABP**(*ω*) and **[HbT]**(*ω*) amplitude ratios and phase differences at each frequency that featured coherent oscillations, averaged over all twenty-two subjects.

**Table 2:** Frequency of hemodynamic oscillations, number of subjects, *n*, whose data showed coherence, group average and standard error for amplitude ratios and phase differences between [HbT](*ω*) and ABP(*ω*).

Frequency (Hz)	*n*	$\frac{\text{|[HbT]|}}{\text{|ABP|}}(\mu M/mmHg)$	∠[HbT] − ∠ABP (deg)
0.040	18	0.101 ± 0.013	-19 ± 3
0.053	22	0.096 ± 0.010	-23 ± 3
0.067	22	0.085 ± 0.009	-31 ± 3
0.080	22	0.074 ± 0.008	-37 ± 3
0.093	22	0.067 ± 0.007	-41 ± 3
0.106	22	0.062 ± 0.006	-47 ± 4
0.120	22	0.061 ± 0.007	-49 ± 4
0.133	22	0.058 ± 0.007	-52 ± 4
0.146	21	0.054 ± 0.008	-54 ± 4
0.160	22	0.050 ± 0.005	-55 ± 4
0.173	22	0.047 ± 0.004	-54 ± 4
0.186	21	0.043 ± 0.005	-53 ± 5

**Fig 7:**
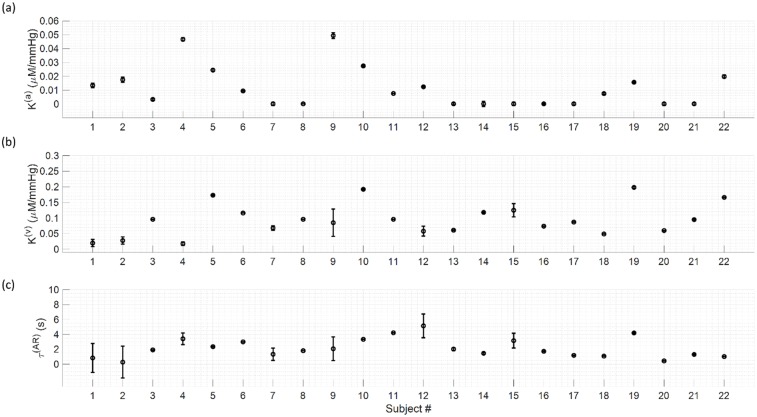
Results for the fitting parameters for all twenty-two subjects. (a): *K*^(*a*)^; (b): *K*^(*v*)^; (c): *τ*^(AR)^.

The best fits of the data with the model of Eq (14) are shown in Fig 6 for the eight representative subjects. The parameter values obtained from the fits are reported in Fig 7 for all twenty-two subjects. Over all subjects, *K*^(*a*)^ ranged from 0.0001 to 0.05 μM/mmHg with a mean value of 0.01 ± 0.01 μM/mmHg; *K*^(*v*)^ ranged from 0.02 to 0.20 μM/mmHg with a mean value of 0.09 ± 0.05 μM/mmHg; *τ*^(AR)^ ranged from 0.3 to 5.1 s with a mean value of 2.2 ± 1.3 s.

**Fig 8:**
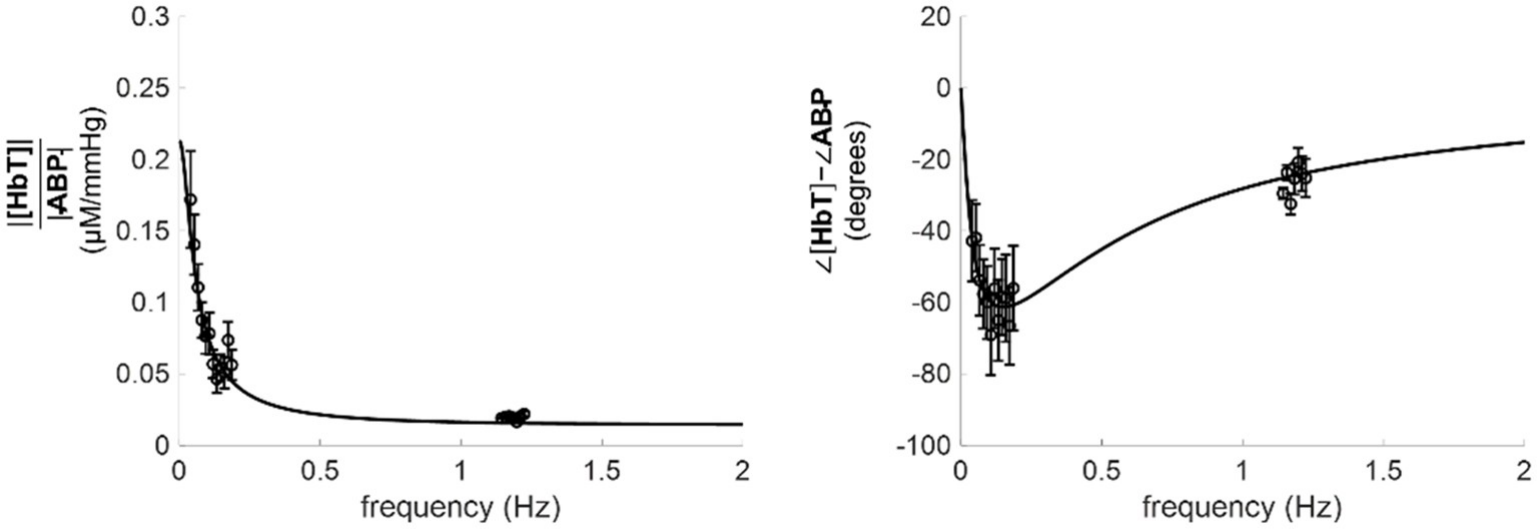
Extension of the results for subject 19 to higher frequencies, up to 2 Hz. Specifically, the fit lines of Fig 6(g), which result from the fit to the data at frequencies below 0.2 Hz, are extended up to a frequency of 2 Hz. The additional experimental data points at about 1.2 Hz represent cerebral [HbT] and ABP oscillations at the heart rate.

The model that we have derived to represent the ABP-[HbT] transfer function [Eq (14)] predicts that at high frequencies (*ω* ≫ 1/*τ*^(AR)^), where cerebral autoregulation is not effective, [HbT] and ABP oscillations tend to be in phase with each other, and with a relative amplitude given by *K*^(*a*)^. The mean value of about 2 s for *τ*^(AR)^ signifies that the condition *ω* ≫ 1/*τ*^(AR)^ is satisfied at the heartbeat frequency of about 1 Hz (in this case *ωτ*^(AR)^ ≈ 4*π* ≫ 1), consistent with the fact that cerebral autoregulation is not effective at the heartbeat frequency. In fact, the extension of the fit lines of Fig 6 to higher frequencies tend to *K*^(*a*)^ for the amplitude ratio, and to zero for the phase difference. We report such extension of the fit lines to higher frequencies in Fig 8 for subject 19, where we also show the amplitude ratio and the phase difference of [HbT] and ABP coherent oscillations measured at the heartbeat frequency (~1.2 Hz). We observed an excellent agreement between the extrapolated fit lines and the observed experimental data at the heart rate around 1.2 Hz.

### 3.2 Spatial mapping probe

**Fig 9:**
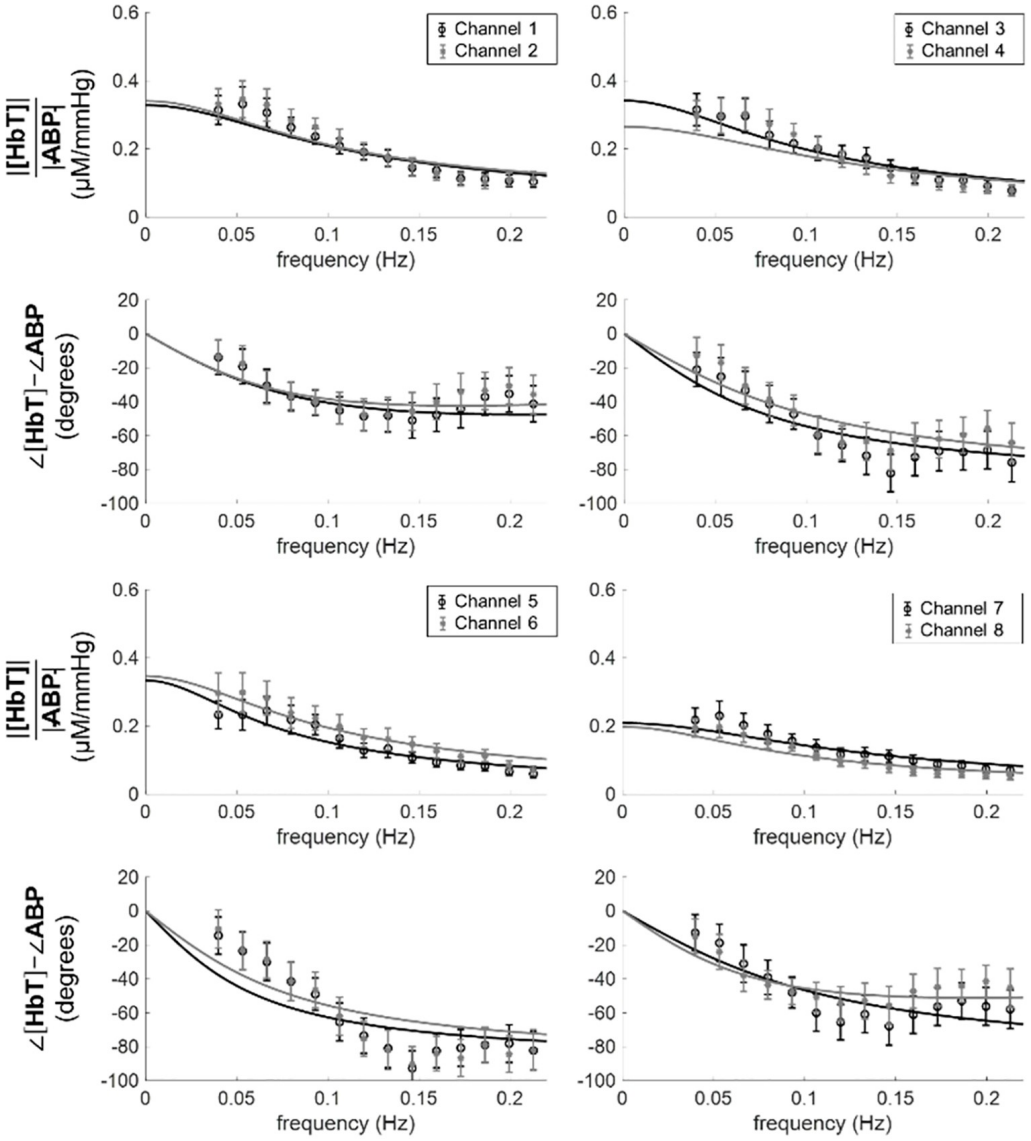
Experimental spectra (symbols) and best fits (lines) with Eq (14) for all channels measured with the spatial mapping probe on subject 5.

Eight of the subjects wore a spatial mapping probe that covered eight locations across the prefrontal cortex. The transfer function between ABP and [HbT] was calculated for each of the channels and a fit to the model of Eq (14) was performed. Fig 9 shows the experimental data for the spectra of all eight channels measured on subject 5, and the lines are the best fit of the model to the data.

**Fig 10:**
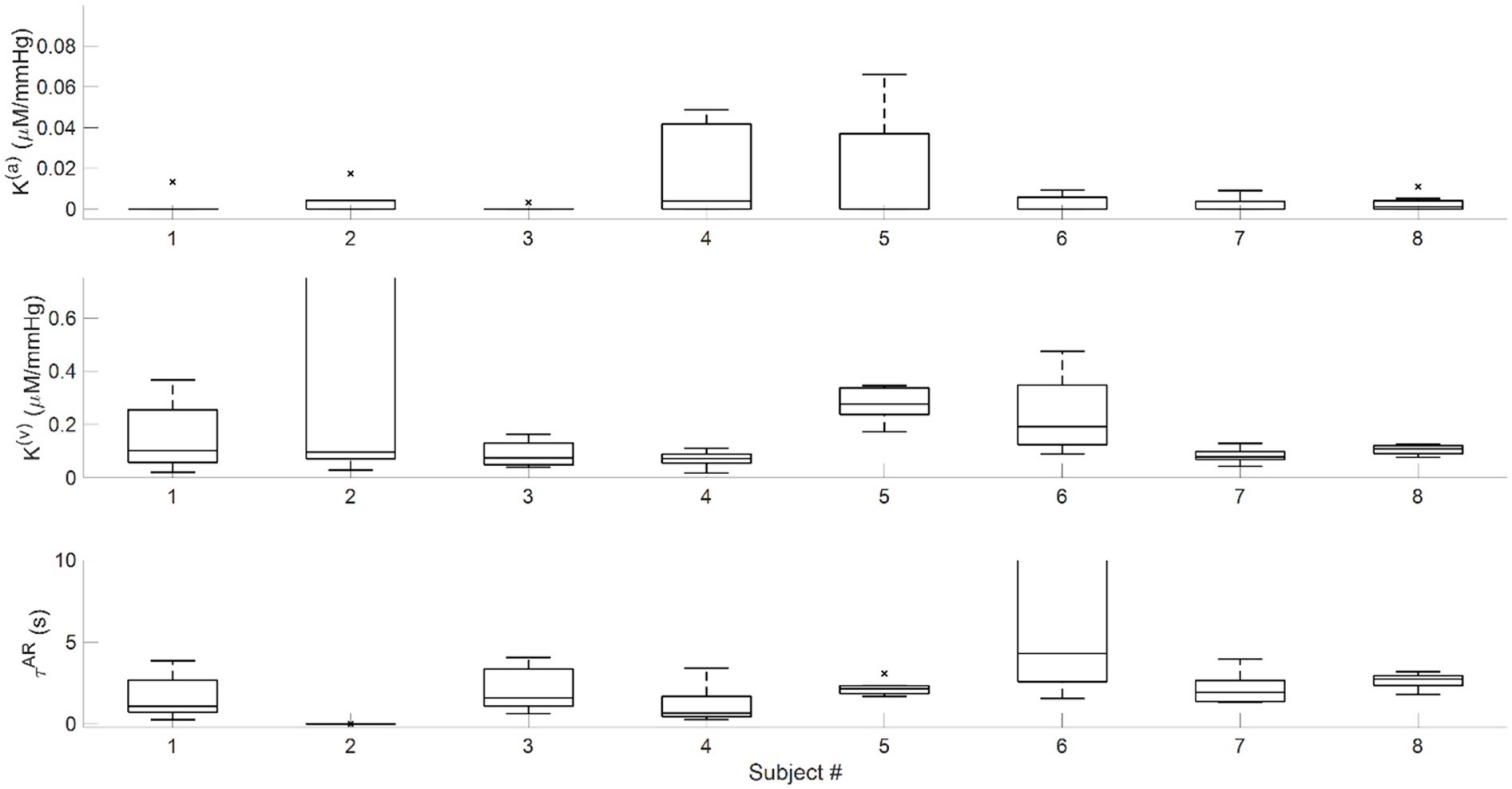
Box plots for the eight subjects measured with the spatial mapping probe. Each box represents the spread of parameters across the eight channels in the probe. Top panel: *K*^(*a*)^. Middle panel: *K*^(*v*)^. Bottom panel: *τ*^(AR)^.

Fig 10 reports the parameter values with box plots for the eight channels in all eight subjects measured with the spatial mapping probe. The central line in each rectangle is the median parameter value across the eight channels. The bottom edge is the 25^th^ percentile and the top edge is the 75^th^ percentile. The whiskers extend to the most extreme data points that are within ±2.7 standard deviations of the mean value. Points beyond the whiskers are considered outliers and are marked with an “x”. The size of the 25^th^-75^th^ percentile interval for the three fitted parameters is highly variable across the eight subjects, and in some cases it indicates a strong spatial dependence. Such spatial dependence may be further exploited for mapping applications, and the associated variance in the measured quantities is a normative reference that would need to be taken into account even in single-location measurements.

## 4. Discussion

We have applied an STFT approach for coherence, amplitude, and phase analysis of non-stationary systemic ABP and cerebral total hemoglobin concentration in the prefrontal cortex of human subjects. Previously, we have used the Hilbert transform-based analytic signal approach for the study of hemodynamic oscillations [40,41]. The benefit of the STFT approach is that it does not require band pass filtering at specific frequencies, which is a time consuming process. Instead, one may consider the data collected over the entire duration of the experiment, and use coherence thresholding to identify coherent dynamics that are associated with both induced and spontaneous ABP oscillations. Analysis of coherence and phase in the time-frequency domain has been used in several studies for analyzing systemic and local signals related to the vascular system [24,25,42–44]. In particular, NIRS-based studies of autoregulation have previously used the wavelet approach for time-frequency analysis [45].

Our results are consistent with the model of Eq (14), which assigns the observed [HbT] dynamics to two sources: one that is synchronous with ABP and results from an arterial compliance response (we neglect here the fast compliance response time, associated with characteristic frequencies of the order of 5 Hz [46]), and one that is lagging ABP and results from a venous volume response to CBF dynamics. In the autoregulated brain, CBF oscillations lead ABP oscillations, but the π/2 phase lag of venous blood volume with respect to CBF (see Eq (9)) results in a net lag of CBV^(*v*)^ vs. ABP. The relative contributions of these two sources to the observed [HbT] dynamics depends on frequency: at high frequencies (*ω* ≫ 1/*τ*^(*AR*)^, or frequencies greater than ~1 Hz since we found *τ*^(*AR*)^ ≈ 2.2 s), the arterial term dominates and accounts for the asymptotic values for amplitude and phase (*K*^(*a*)^ and 0, respectively) that one can see in Fig 8. At low frequencies (*ω* ≲ 1/*τ*^(*AR*)^, or frequencies lower than ~0.1 Hz), the venous term dominates and the transfer function *H*_ABP,[HbT]_(*ω*) takes the form of a low-pass filter. According to this model, in this low-frequency range (≲ 0.1 Hz) the CBF phase is given by the [HbT] phase + π/2. This observation can be the basis for a novel NIRS-based approach to the dynamic study of CBF. Specifically, in this low-frequency range, this π/2 phase shift may be applied to measured [HbT] oscillations in order to determine the phase of CBF oscillations.

According to our interpretation, [HbT] dynamics measured with NIRS are mostly determined by passive arterial responses to ABP dynamics and passive venous responses to CBF dynamics. Therefore, active arteriolar responses to ABP changes would not be directly measurable with NIRS, but they would be indirectly measurable through the effects of CBF on CBV^(*v*)^. The fact that [HbT] is insensitive to the active dilation and constriction of arterioles may result from arteriolar contributions to the NIRS-measured [HbT] that are overwhelmed by contributions from larger arteries and from veins. Of course, [HbT] may also be significantly linked to intracranial pressure (ICP), which we have not monitored in this study. ICP is relatively constant in healthy individuals, but the effect of ICP may become important in the case of patients with cerebrovascular pathologies.

While our interpretation of the phase lag of [HbT] vs. ABP is related to the CBF dynamics and the π/2 phase lag of venous blood volume with respect to CBF oscillations, one may also consider a potential role of the propagation time of arterial blood pressure and the blood transit time in the cerebral microvasculature. Even though we measure ABP on the subject’s finger, the propagation time of arterial pressure waves from the macrovasculature to the microvasculature is of the order of 10–100 μs [47,48], indicating that its contribution to our observed phase lag, at least for frequencies ≲ 0.1 Hz, is negligible. The blood transit time through the microvasculature may provide a more sizeable contribution. The arteriovenous transit time (AVTT) from a branch of the middle cerebral artery (MCA) to a draining vein in the mice cortex was found to be in the range 0.35–1.18 s using videomicroscopy and intravascular tracers [49]. This blood transit time may provide some contribution to the observed phase lag of [HbT] vs. ABP. As a point of reference, a fixed delay of 1 s (*τ*_*d*_ = 1 s) would contribute a frequency-dependent phase lag (*ωτ*_*d*_) that takes a value of 18° at 0.1 Hz.

The mean value of *K*^(*a*)^ (0.01 ± 0.01 μM/mmHg) was significantly lower than the mean value of *K*^(*v*)^ (0.09 ± 0.05 μM/mmHg), indicating a smaller change in arterial volume than in venous volume (in the low-frequency limit, since the venous volume change decreases with frequency). The observed variability in *K*^(*a*)^ and *K*^(*v*)^ may reflect differences in the relative arterial and venous blood volumes across subjects and over different anatomical locations. Such variability, which may carry valuable physiological information in and of itself, would need to be further characterized, for example by comparing inter-subject variations (as reported here) with intra-subject variations (at different times, at different cortical locations, under different physiological/functional conditions, etc.). The relative arterial and venous contributions to hemodynamic changes associated with functional or physiological processes is an area of active research [50–52]. Larger arteries respond to changes in transmural pressure, smaller arterioles actively modulate their diameter to regulate blood flow, and venules passively respond to changes in pressure and flow. However, the partial arterial contribution to blood volume (~30% [53]) may be further reduced in dynamic studies as a result of the much more compliant nature of veins [54]. Baker *et al*. studied arterial vessel compliance in the microvasculature with DCS [55]. We have translated the compliance values they found into units of hemoglobin concentration per unit arterial blood pressure, which resulted in a one-standard-deviation range of 0.005-0.018 μM/mmHg. These values are in agreement with our results for *K*^(*a*)^.

In our simplified model of autoregulation (based on Eq (10) with the impulse response function of a first-order high pass filter), we introduce a time constant for autoregulation (*τ*^(AR)^), which quantifies the frequency dependence of CBF on ABP. This time constant corresponds to a characteristic cutoff frequency of autoregulation given by $f_{c}^{\left(AR\right)}=1/(2\pi \tau^{\left(AR\right)})$. A higher cutoff frequency indicates a more efficient autoregulation, because it signifies an attenuation of CBF oscillations over a broader range of frequencies. We found mean values of *τ*^(AR)^ and $f_{c}^{\left(AR\right)}$ of 2.2 ± 1.3 s and 0.07 ± 0.4 Hz, respectively. Kainerstorfer *et al*. [28] translated the results of Aaslid *et al*. [56] to compute a cutoff frequency of AR between 0.03 to 0.06 Hz, dependent upon arterial CO_2_ concentration. Fraser *et al*. found similar values for the cutoff frequency of autoregulation with a range between 0.025 to 0.036 Hz [57]. Our result of 0.07 ± 0.4 Hz is somewhat greater, possibly as a result of a different nature of our measurements, a different mathematical framework for our analysis, and the neglected potential role of microvascular blood transit times.

Previous studies have investigated the relative phase of oscillations of hemoglobin concentration and arterial blood pressure. In some cases, MAP was reported to lead [HbO_2_] [14,15] in agreement with our results, while in other cases oscillations in [HbO_2_] were found to lead those in MAP at frequencies with high wavelet coherence [58]. These latter results are at odds with ours, but this discrepancy may be accounted for by the large variability of the estimated phase in [58]. Payne *et al*. presented models for the transfer functions of [HbO_2_] vs ABP and [Hb] vs ABP [59]. The models considered arterial, capillary, and venous compartments, with arterial and venous compartments behaving like balloons, and incorporating equations for oxygen transport based on the concentrations of oxyhemoglobin and deoxyhemoglobin. Their model indicated that the ratio of arterial to venous blood volume has a strong influence on phase dynamics. The frequency domain transfer function of [HbO_2_] and ABP, which they simulated with nominal parameters, exhibited a similar low pass filter relationship that we have found in our experimental data. Payne *et al*. compared their results to those of Reinhard *et al*. [15], who reported phase delays at a single frequency of 0.1 Hz, because experimental data for phase delays at multiple frequencies were not available at that time. Payne *et al*. found an agreement with the results of Reinhard *et al*. Quick *et al*. also studied the frequency-dependent relationship between volume, derived from CBF, and pressure, and similarly found a low-pass relationship [46,60].

In our experimental study and theoretical analysis of the dynamic relationship between [HbT] and ABP, we stressed the importance of frequency, which is known to impact the role of vascular resistance and compliance in dynamic autoregulation [61]. The transfer function for [HbT] and ABP can be impacted by a number of factors; for example, the concentration of arterial blood gases such as carbon dioxide [62–64], cerebral spinal fluid and global compartment compliance [65,66], the effects of the arterial compartment on the venous compartment [54], properties of absolute arterial pressure (baseline value, direction of change, rate of change) [67], baroreceptor sensitivity [66], disorders of the carotids or other large arteries [68,69], low global cerebral oxygen metabolism [70], and altered arterial compliance [71].

The spatial variability observed in the eight subjects investigated with the spatial mapping probe provides normative data for future imaging and single-location studies. A NIRS spatial mapping approach has already shown utility in observing hemispheric differences in patients with unilateral carotid disease [72]. In that study, the phase between [HbO_2_] and ABP was computed for oscillations at 0.1 Hz, and a diagnostic cutoff value of -50° was considered. In this perspective, it is important to characterize the intrinsic phase variance due to anatomical heterogeneity at different cortical locations.

In our NIRS measurements, we used a source-detector distance of 3.5 cm, which is well-established to achieve optical sensitivity to the cerebral cortex. However, the nature of noninvasive NIRS measurements is such that they are also sensitive to extracerebral tissue layers (scalp, skull, dura, arachnoid/subarachnoid space, etc.). Preliminary measurements of coherent hemodynamics driven by arterial blood pressure oscillations reveal a small dependence of the relative phase of [HbT] and ABP oscillations on source-detector separation, thus supporting the validity of cortical measurements of [HbT] dynamics at a single source-detector distance of 3.5 cm [73].

The chirp-like protocol for inducing cerebral hemodynamics with cyclic inflation and deflation of thigh cuffs can cover the same number of frequencies in less time than the spaced oscillation approach. Our initial results indicate a strong potential for the faster chirp-like experimental protocol, which has a number of practical advantages associated with its shorter duration. In this study, we assumed that the timing and duration of ABP oscillations in different protocols does not affect the transfer function between ABP and [HbT]. In our experience, [HbT] and ABP oscillations that are coherent with each other feature similar phase relationships under a variety of protocols, including spontaneous oscillations at baseline, and sequential or chirp-like induced oscillations. However, the insensitivity of the relative dynamics of coherent [HbT] and ABP oscillations is an important point that we intend to further validate and demonstrate in future studies.

## 5. Conclusions

We have reported a human study of the dynamic relationship between cerebral hemoglobin concentration (measured with NIRS in the prefrontal cortex) and systemic arterial blood pressure (measured with finger plethysmography). Specifically, we generated a theoretical transfer function to describe the frequency-dependent relationship between [HbT] and ABP oscillations, and we validated it experimentally in the frequency range 0.04–0.20 Hz, as well as at the heart rate (~1 Hz). Our results are consistent with two mechanisms of [HbT] oscillations, one directly due to ABP oscillations (affecting the arterial compartment and dominating at high frequencies beyond ~1 Hz) and one due to ABP-driven oscillations in CBF (affecting the venous compartment and dominating at low frequency below ~0.1 Hz). According to this interpretation, NIRS measurements of [HbT] reflect the autoregulatory CBF response, at least in the low-frequency range of about 0.1 Hz or below. Consequently, this study sets the stage for a new approach to optical measurements of cerebral autoregulation, on the basis of robust measurements of [HbT] and ABP dynamics.

## Supporting information

S1 Dataset Signals database for subject 1. Time traces of pneumatic cuff pressure, heart rate, arterial blood pressure, Δ[HbT] and ABP-[HbT] transfer functions for eight channels.
(XLSX)

S2 Dataset Signals database for subject 2. Time traces of pneumatic cuff pressure, heart rate, arterial blood pressure, Δ[HbT] and ABP-[HbT] transfer functions for eight channels.
(XLSX)

S3 Dataset Signals database for subject 3. Time traces of pneumatic cuff pressure, heart rate, arterial blood pressure, Δ[HbT] and ABP-[HbT] transfer functions for eight channels.
(XLSX)

S4 Dataset Signals database for subject 4. Time traces of pneumatic cuff pressure, heart rate, arterial blood pressure, Δ[HbT] and ABP-[HbT] transfer functions for eight channels.
(XLSX)

S5 Dataset Signals database for subject 5. Time traces of pneumatic cuff pressure, heart rate, arterial blood pressure, Δ[HbT] and ABP-[HbT] transfer functions for eight channels.
(XLSX)

S6 Dataset Signals database for subject 6. Time traces of pneumatic cuff pressure, heart rate, arterial blood pressure, Δ[HbT] and ABP-[HbT] transfer functions for eight channels.
(XLSX)

S7 Dataset Signals database for subject 7. Time traces of pneumatic cuff pressure, heart rate, arterial blood pressure, Δ[HbT] and ABP-[HbT] transfer functions for eight channels.
(XLSX)

S8 Dataset Signals database for subject 8. Time traces of pneumatic cuff pressure, heart rate, arterial blood pressure, Δ[HbT] and ABP-[HbT] transfer functions for eight channels.
(XLSX)

S9 Dataset Signals database for subject 9. Time traces of pneumatic cuff pressure, heart rate, arterial blood pressure, Δ[HbT] and ABP-[HbT] transfer function for one channel.
(XLSX)

S10 Dataset Signals database for subject 10. Time traces of pneumatic cuff pressure, heart rate, arterial blood pressure, Δ[HbT] and ABP-[HbT] transfer function for one channel.
(XLSX)

S11 Dataset Signals database for subject 11. Time traces of pneumatic cuff pressure, heart rate, arterial blood pressure, Δ[HbT] and ABP-[HbT] transfer function for one channel.
(XLSX)

S12 Dataset Signals database for subject 12. Time traces of pneumatic cuff pressure, heart rate, arterial blood pressure, Δ[HbT] and ABP-[HbT] transfer function for one channel.
(XLSX)

S13 Dataset Signals database for subject 13. Time traces of pneumatic cuff pressure, heart rate, arterial blood pressure, Δ[HbT] and ABP-[HbT] transfer function for one channel.
(XLSX)

S14 Dataset Signals database for subject 14. Time traces of pneumatic cuff pressure, heart rate, arterial blood pressure, Δ[HbT] and ABP-[HbT] transfer function for one channel.
(XLSX)

S15 Dataset Signals database for subject 15. Time traces of pneumatic cuff pressure, heart rate, arterial blood pressure, Δ[HbT] and ABP-[HbT] transfer function for one channel.
(XLSX)

S16 Dataset Signals database for subject 16. Time traces of pneumatic cuff pressure, heart rate, arterial blood pressure, Δ[HbT] and ABP-[HbT] transfer function for one channel.
(XLSX)

S17 Dataset Signals database for subject 17. Time traces of pneumatic cuff pressure, heart rate, arterial blood pressure, Δ[HbT] and ABP-[HbT] transfer function for one channel.
(XLSX)

S18 Dataset Signals database for subject 18. Time traces of pneumatic cuff pressure, heart rate, arterial blood pressure, Δ[HbT] and ABP-[HbT] transfer function for one channel.
(XLSX)

S19 Dataset Signals database for subject 19. Time traces of pneumatic cuff pressure, heart rate, arterial blood pressure, Δ[HbT] and ABP-[HbT] transfer function for one channel.
(XLSX)

S20 Dataset Signals database for subject 20. Time traces of pneumatic cuff pressure, heart rate, arterial blood pressure, Δ[HbT] and ABP-[HbT] transfer function for one channel.
(XLSX)

S21 Dataset Signals database for subject 21. Time traces of pneumatic cuff pressure, heart rate, arterial blood pressure, Δ[HbT] and ABP-[HbT] transfer function for one channel.
(XLSX)

S22 Dataset Signals database for subject 22. Time traces of pneumatic cuff pressure, heart rate, arterial blood pressure, Δ[HbT] and ABP-[HbT] transfer function for one channel.
(XLSX)
